# Antinociceptive Activity of Methanolic Extract of *Clinacanthus nutans* Leaves: Possible Mechanisms of Action Involved

**DOI:** 10.1155/2018/9536406

**Published:** 2018-03-04

**Authors:** Zainul Amiruddin Zakaria, Mohammad Hafiz Abdul Rahim, Rushduddin Al Jufri Roosli, Mohd Hijaz Mohd Sani, Maizatul Hasyima Omar, Siti Farah Mohd. Tohid, Fezah Othman, Siew Mooi Ching, Arifah Abdul Kadir

**Affiliations:** ^1^Department of Biomedical Science, Faculty of Medicine and Health Sciences, Universiti Putra Malaysia, 43400 UPM Serdang, Selangor, Malaysia; ^2^Integrative Pharmacogenomics Institute (iPROMISE), Faculty of Pharmacy, Universiti Teknologi MARA, Puncak Alam Campus, 42300 Bandar Puncak Alam, Selangor, Malaysia; ^3^Phytochemistry Unit, Herbal Medicine Research Centre, Institute for Medical Research, Jalan Pahang, 50588 Kuala Lumpur, Malaysia; ^4^Department of Family Medicine, Faculty of Medicine and Health Science, Universiti Putra Malaysia, 43400 Serdang, Selangor, Malaysia; ^5^Department of Veterinary Pre-Clinical Sciences, Faculty of Veterinary Sciences, Universiti Putra Malaysia, 43400 Serdang, Selangor, Malaysia

## Abstract

Methanolic extract of *Clinacanthus nutans* Lindau leaves (MECN) has been proven to possess antinociceptive activity that works via the opioid and NO-dependent/cGMP-independent pathways. In the present study, we aimed to further determine the possible mechanisms of antinociception of MECN using various nociceptive assays. The antinociceptive activity of MECN was (i) tested against capsaicin-, glutamate-, phorbol 12-myristate 13-acetate-, bradykinin-induced nociception model; (ii) prechallenged against selective antagonist of opioid receptor subtypes (*β*-funaltrexamine, naltrindole, and nor-binaltorphimine); (iii) prechallenged against antagonist of nonopioid systems, namely, *α*_2_-noradrenergic (yohimbine), *β*-adrenergic (pindolol), adenosinergic (caffeine), dopaminergic (haloperidol), and cholinergic (atropine) receptors; (iv) prechallenged with inhibitors of various potassium channels (glibenclamide, apamin, charybdotoxin, and tetraethylammonium chloride). The results demonstrated that the orally administered MECN (100, 250, and 500 mg/kg) significantly (*p* < 0.05) reversed the nociceptive effect of all models in a dose-dependent manner. Moreover, the antinociceptive activity of 500 mg/kg MECN was significantly (*p* < 0.05) inhibited by (i) antagonists of μ-, *δ*-, and *κ*-opioid receptors; (ii) antagonists of *α*_2_-noradrenergic, β-adrenergic, adenosinergic, dopaminergic, and cholinergic receptors; and (iii) blockers of different K^+^ channels (voltage-activated-, Ca^2+^-activated, and ATP-sensitive-K^+^ channels, resp.). In conclusion, MECN-induced antinociception involves modulation of protein kinase C-, bradykinin-, TRVP1 receptors-, and glutamatergic-signaling pathways; opioidergic, *α*_2_-noradrenergic, *β-*adrenergic, adenosinergic, dopaminergic, and cholinergic receptors; and nonopioidergic receptors as well as the opening of various K^+^ channels. The antinociceptive activity could be associated with the presence of several flavonoid-based bioactive compounds and their synergistic action with nonvolatile bioactive compounds.

## 1. Introduction

Pain, an unpleasant experience caused by intense or damaging stimuli, is primarily protective in nature and can act as a sensorial modality to indicate the presence of tissues injury [1, 2]. There are various types and causes of pain, but all relate to a sensation of physical or emotional discomfort that affects daily routine negatively. Despite efforts to relieve pain, harmful physiological effects can ensue, including inadequate sleep, exhaustion, disorientation, anxiety, tachycardia, increased myocardial oxygen demand, immunosuppression, and increased catabolism [3].

The management of pain using currently available analgesics could not completely thrive in relieving pain due to the fact that pain modulation is an intricate process involving many mediators and receptors at the peripheral and central levels. The sensitivity of nociceptive neuron is adjusted by a large variety of mediators in the extracellular space. These mediators, either neurotransmitters or neuromodulators, activate a large number of receptor classes, which in turn activate a plethora of signaling cascades that are responsible for controlling the perception of pain [4–7]. How this multitude of cascades mediates nociceptor sensitization and pain is only beginning to be understood. Thus, attempts are being made worldwide to identify the components involved in this complex process and to develop new agents that act on these components [1]. Other than that, currently available analgesic drugs such as opiates and nonsteroidal antiinflammatory drugs (NSAIDs) are not useful in all cases as their effectiveness have been over shadowed by various adverse effects [2]. For example, morphine, which has been the drug of choice for the treatment of pain, has been known to cause dependence and tolerance upon its prolonged usage [8]. Further worsening the situation, available analgesics relieve pain as a symptom without affecting its cause [9]. Therefore, search for new analgesic drugs with promising pharmacological actions has become an urgent need.

Medicinal plants are believed to be an important source of new chemical substances with potential therapeutic effects [10]. Different parts of the medicinal plants have been utilized for various therapeutic purposes in folk medicine. Indeed, many of the plants and their preparations have been recorded to be used to relieve pain and promote healing. Therefore, research in medicinal plants possessing a wide diversity of phytochemicals in terms of analgesic/antinociceptive activities seems to be necessary and beneficial. The need to search and study phytoconstituents with antinociceptive activity is further warranted given the fact that current analgesic drugs produced by chemical synthesis have potential side effects. Moreover, the discovery of plant-based medications with high therapeutic efficacy, but fewer or, possibly, no side effects for pain management, might be of beneficial interest as replacements to conventional analgesics like opiates and NSAIDs [11].

One of the medicinal plants that are currently being investigated for its potential to relieve pain is *Clinacanthus nutans* (*C. nutans*) Lindau. This herb, locally known as “Belalai gajah,” is a small shrub belonging to the family Acanthaceae and can be found in the tropical Southeast Asian countries, including Malaysia. The plant is traditionally used by the local communities in Malaysia, Indonesia, and Thailand to treat various types of ailments while, scientifically, the plant has been reported to demonstrate various pharmacological activities, including antinociceptive activity [12]. Earlier study has proven that the methanol extract of *C. nutans* (MECN) possesses antinociceptive activity at the peripheral and central levels. Moreover, the study also revealed the mechanisms of antinociception of MECN, which involved activation of the opioid receptors and modulation of the nitric oxide-mediated but cGMP-independent pathways. Phytochemical analysis of MECN using the UHPLC-ESI and GCMS methods also demonstrated the presence of various nonvolatile and volatile bioactive compounds, of which, some have been reported to exert antinociceptive activity [12]. It is believed that that these compounds might act synergistically to exhibit the antinociceptive activity.

Given the fact that (i) pain transmission is a complex process that involves activation of a plethora of signaling cascades by various mediators through numerous receptors at the peripheral and central levels and (ii) currently available analgesics are associated with adverse effects that may overshadowed their effectiveness, the present study was carried out with an aim of further elucidating the mechanisms of antinociception exerted by MECN using various nociceptive models in mice.

## 2. Materials and Methods

### 2.1. Plant Collection

Fresh *C. nutans* leaves were obtained from Clinnthus Enterprise (Kuala Lumpur, Malaysia) in January 2013. Authentication of the plant was made by Dr. Shamsul Khamis, a botanist from the Institute of Bioscience, Universiti Putra Malaysia (UPM), Serdang, Selangor, Malaysia, and a voucher specimen (SK 2679/15) has been deposited at the herbarium of the institute.

### 2.2. Preparation of MECN

Extraction was carried out according to the method described previously [12]. To obtain the MECN, 250 g of *C. nutans* leaves, which were dried in an oven at 40°C for 1-2 days and grounded into powder form by using an electric grinder (RT-08; Rong Tsong Precision Technology, Taichung, Taiwan), were soaked in methanol (Fisher Scientific, Loughborough, England) in the ratio of 1 : 20 (w/v) for 72 hours at room temperature. The supernatant was filtered by using a steel filter, cotton wool, and Whatman Number 1 filter paper. The residue underwent the same soaking procedures twice. The supernatant collection from each of the extractions was pooled and evaporated using a vacuum rotary evaporator (Hei-VAP Value; Heidolph, Schwabach, Germany) at 40°C under reduced pressure. These processes yielded approximately 53 g of dried MECN (yield was 21.2% (w/w)), which was then stored at 4°C until used.

### 2.3. Experimental Animals

The antinociceptive studies were carried out using adult male ICR mice (25–30 g), which were obtained from the Animal Source Unit, Faculty of Veterinary Medicine, UPM, Serdang, Malaysia. The animals were kept at room temperature (27 ± 2°C; 70–80% humidity; 12 h light/dark cycle) in the Animal Holding Unit, Faculty of Medicine and Health Science, UPM, for at least 48 h prior to the procedure. Commercial food pellets (Gold Coin Feed Mills, Port Klang, Malaysia) and water were supplied *ad libitum*. The animal experimental protocols were in accordance with the current guidelines for the care of laboratory animals and the ethical guidelines for investigations of experimental pain in conscious animals as adopted from Zimmermann [13] and have been approved by the UPM Institutional Animal Care and Use Committee (Ref. Number UPM/IACUC/AUP-R032/2013). The number of animals and intensities of noxious stimuli used were the minimum necessary to demonstrate the consistent effects of the treatments. Experiments were conducted between 0930 h and 1830 h to minimize the effects of environmental changes.

### 2.4. Drugs and Chemicals

The following drugs were used: (i) acetylsalicylic acid (ASA), apamin, atropine, bradykinin, caffeine, capsaicin, capsazepine (CAPZ), charybdotoxin, glibenclamide, haloperidol, l-glutamic acid, phorbol 12-myristate 13-acetate (PMA), pindolol, tetraethylammonium chloride, and yohimbine were purchased from Sigma-Aldrich (St. Louis, MO, USA); (ii) naltrindole hydrochloride, nor-binaltorphimine dihydrochloride and *β*-funaltrexamine hydrochloride were purchased from Tocris Bioscience (Ellisville, Missouri, USA); and (iii) acetic acid, dimethyl sulfoxide (DMSO), and methanol were purchased from Fisher Scientific (England). Bradykinin, capsaicin, l-glutamic acid, and PMA were dissolved in physiological saline (0.9% (w/v) NaCl), while ASA, MECN, and CAPZ were dissolved in distilled water containing 10% DMSO (v/v). The vehicle used alone had no effects per se on the nociceptive responses in mice. All drugs, chemicals, and MECN solutions were administered in 10 mL/kg volumes and were freshly prepared just before being used.

### 2.5. Antinociceptive Effect of MECN against Capsaicin-, Glutamate-, Phorbol 12-Myristate 13-Acetate- (PMA-), and Bradykinin-Induced Nociception

The protocol used was similar to the method previously described [14–17]. For that purpose, the mice (*n*=6) were treated with vehicle (10 mL/kg, p.o.), MECN (100, 250, and 500 mg/kg, p.o.), CAPZ (TRPV1 antagonist; 0.17 mmol/kg, p.o.; served as the positive control for capsaicin test), or ASA (100 mg/kg; served as the positive control for glutamate, PMA, and bradykinin tests) 60 mins before they were injected (20 *μ*L) with capsaicin (1.6 *μ*g/paw), glutamate (10 umol/paw), PMA (a protein kinase C activator; 0.05 *μ*g/paw), or bradykinin (10 nmol/paw), respectively, into the intraplantar (i.pl) route the ventral surface of right hind paw. Immediately after the administration of the phlogistic agents, the animals were observed individually in the transparent glass cage observation chamber from 0 to 5 min (capsaicin), 0 to 15 min (glutamate), 15 to 45 min (PMA), or 0 to 10 min (bradykinin), respectively. The amount of time the mice spent licking the injected paw was recorded using a chronometer and was considered as an indicative of nociception.

### 2.6. Involvement of Nonopioid and Opioid Systems in the Antinociceptive Activity of MECN

The possible roles of nonopioid and opioid receptor antagonists were performed as previously described [18]. For nonopioid receptor antagonists, the mice (*n*=6) were pretreated with yohimbine (YOH; 0.15 mg/kg, i.p.), pindolol (PDL; 1 mg/kg, i.p.), caffeine (CAF; 3 mg/kg, i.p.), haloperidol (HAL; 0.2 mg/kg, i.p.), or atropine (ATR; 10 mg/kg, i.p.) 15 mins before administration of vehicle (10 mL/kg, p.o.) or MECN (500 mg/kg, p.o.). In separate experiments, for opioid receptor antagonists, the *μ* opioid antagonist, *β*-funaltrexamine (*β*FNA; 10 mg/kg, i.p.), *δ* opioid receptor antagonist, naltrindole (NALT; 1 mg/kg, i.p.) or *κ* opioid receptor antagonist, nor-binaltorphimine (nor-BNI; 1 mg/kg, i.p.) were administered 90 min, 15 min, and 30 min, respectively, before administration of vehicle (10 mL/kg, p.o.) or MECN (500 mg/kg, p.o.). Sixty minutes after the administration of test solutions, the mice were subjected to the acetic acid-induced abdominal writhing test as described previously in detail (Abdul Rahim et al., 2016). The number of writhings was counted cumulatively over the period of 25 min, 5 min following the acetic acid injection.

### 2.7. Involvement of Potassium Channels in the Antinociceptive Activity of MECN

To investigate the possible participation of various potassium channels blockers in the antinociceptive properties of MECN, the mice (*n*=6) were pretreated with glibenclamide (GLIB; an ATP-sensitive K^+^ channel inhibitor; 10 mg/kg, i.p.), apamin (APA; an inhibitor of small conductance Ca^2+^-activated K^+^ channels, 0.04 mg/kg, i.p.), charybdotoxin (CHAR; an inhibitor of large conductance Ca^2+^-activated K^+^ channels, 0.02 mg/kg, i.p.), or tetraethylammonium chloride (TEA; a nonselective voltage dependent K^+^ channel inhibitor, 4 mg/kg, i.p.) 15 mins before oral administration of either vehicle (10 mL/kg) or MECN (500 mg/kg). The doses of the various potassium channel blockers similar to those reported by Alves and Duarte [19] were used in this study. Sixty minutes after the administration of test solutions, mice were subjected to the acetic acid-induced abdominal writhing test as previously described [12]. The number of writhings was counted cumulatively over the period of 25 min, 5 min following acetic acid injection.

### 2.8. HPLC Analysis of MECN

The HPLC analysis of MECN was carried out in the Laboratory of Phytomedicine, Medicinal Plants Division, Forest Research Institute of Malaysia (FRIM), Kepong, Malaysia, according to the method described by Zakaria et al. [20]. A solution containing a suspended mixture of 10 mg MECN in 1 ml water was prepared and filtered through a filter cartridge (pore size of 0.45 *µ*m) prior to analysis. The HPLC system used for analyzing MECN consisted of Waters Delta 600 with 600 Controller equipped with photodiode array detector (Waters 996) and a Phenomenex Luna column (5 *µ*m; 4.6 mm i.d. × 250 mm) (Torrance, CA, USA). Elution of the constituents was achieved using two solvent systems labelled as A (0.1% aqueous formic acid) and B (acetonitrile). Initial conditions were 85% A and 15% B with a linear gradient reaching 25% B at *t*=12 min, and this condition was maintained for 10 min. B was reduced back to 15%, the initial solvent composition, for 2 min (*t*=22 min) and then maintained until *t*=35 min. The flow rate used was 1.0 ml/min, and the injection volume was 10 *µ*l. The column oven was set at 27°C, and the eluent was monitored at 210, 254, 280, 300, 330, and 366 nm. The retention times, peak areas, and UV spectra of the major peaks were analyzed. The chromatogram of MECN obtained from the HPLC analysis was compared against the chromatogram of several pure flavonoid-based bioactive compounds (i.e., fisetin, quercetin, rutin, quercitrin, naringenin, genistein, pinostrobin, hesperetin, and dihydroquercetin) to determine their presence in the fraction.

### 2.9. UHPLC-ESI and GCMS Profiling of MECN

The extract, MECN, has been earlier subjected to the UHPLC-ESI and GCMS analyses, and findings were reported elsewhere [12]. Findings of these analyses are highlighted in Results and discussed in detail in Discussion.

### 2.10. Data Analysis

For the data analysis, the GraphPad Prism version 6.04 for Windows (GraphPad Software, San Diego, CA, USA) was used. Data are expressed as the mean ± standard error of the mean (SEM). The mean differences between the control and treatment groups were determined using the one-way analysis of variance (ANOVA) with Dunnett's post hoc tests or 2-way ANOVA followed by Bonferroni's post-test. In all cases, the differences were considered as significant if *p* < 0.05.

## 3. Results

### 3.1. Effect of MECN on Capsaicin-, Glutamate-, Phorbol 12-Myristate 13-Acetate- (PMA-), and Bradykinin-Induced Nociception

**Figure 1:**
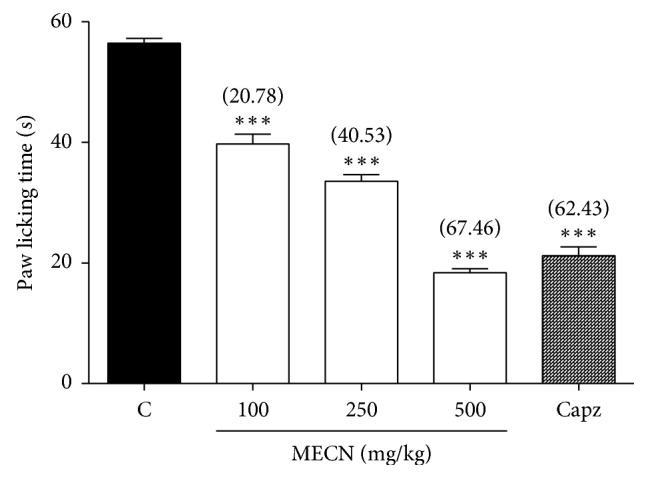
Effect of MECN on capsaicin-induced nociception in mice. Animals were treated with vehicle (10 mL/kg, p.o.), CAPZ (0.17 mmol/kg, p.o.), or MECN (100, 250, and 500 mg/kg, p.o.) 60 min before intraplantar administration of capsaicin (1.6 *μ*g/paw prepared in normal saline; 20 *µ*L) into the right hind paw. Each column represents the mean ± SEM of six mice. Statistical analyses were performed using 1-way ANOVA followed by Dunnett's post hoc test. ^∗∗∗^*p* < 0.001 compared to the control group. Values in parentheses denote percentage of inhibition.

The effect of MECN on capsaicin-induced nociception in mice is shown in Figure 1. The oral administration of MECN (100, 250, and 500 mg/kg) produced significant (*p* < 0.001) and dose-related inhibition of the capsaicin-induced neurogenic pain. MECN at the doses of 100, 250, and 500 mg/kg reduced the paw-licking response by 20.78%, 40.53%, and 67.46%, respectively, compared to the control group. Furthermore, CAPZ (0.17 mmol/kg) which was used as positive control drug showed 62.43% inhibition compared to the control group.

**Figure 2:**
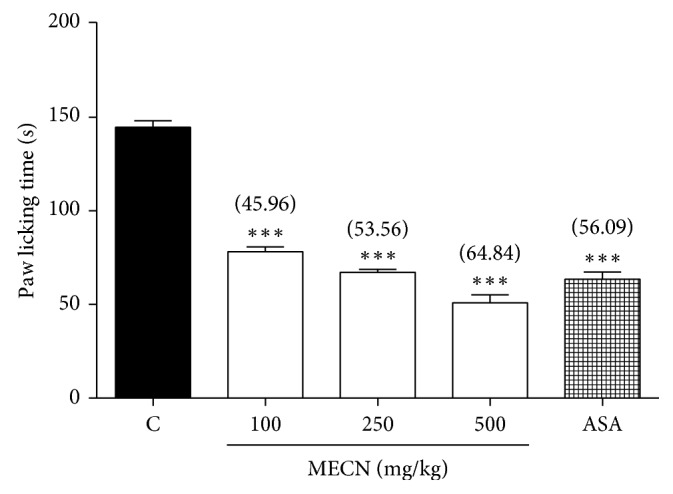
Effect of MECN on glutamate-induced nociception in mice. Animals were treated with vehicle (10 mL/kg, p.o.), ASA (100 mg/kg, p.o.), or MECN (100, 250, and 500 mg/kg, p.o.) 60 min before intraplantar administration of glutamate (10 umol/paw prepared in normal saline; 20 *µ*L) into the right hind paw. Each column represents the mean ± SEM of six mice. Statistical analyses were performed using 1-way ANOVA followed by Dunnett's post hoc test. ^∗∗∗^*p* < 0.001 compared to the control group. Values in parentheses denote percentage of inhibition.

As demonstrated in Figure 2, MECN (100, 250, and 500 mg/kg) produced significant (*p* < 0.001) and dose-related inhibition of glutamate-induced nociception with percentage of inhibition observed at 45.96%, 53.56%, and 64.84%, respectively, when compared to the control group. Moreover, ASA (100 mg/kg) which was used as positive control drug showed 56.09% inhibition as compared to the control group.

**Figure 3:**
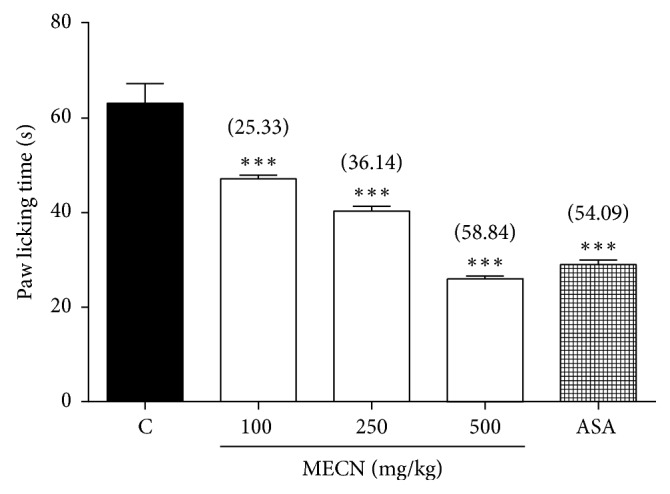
Effect of MECN on PMA-induced nociception in mice. Animals were treated with vehicle (10 mL/kg, p.o.), ASA (100 mg/kg, p.o.), or MECN (100, 250, and 500 mg/kg, p.o.) 60 min before intraplantar administration of glutamate (0.05 *μ*g/paw prepared in normal saline; 20 *µ*L) into the right hind paw. Each column represents the mean ± SEM of six mice. Statistical analyses were performed using 1-way ANOVA followed by Dunnett's post hoc test. ^∗∗∗^*p* < 0.001 compared to the control group. Values in parentheses denote percentage of inhibition.

Using the PMA-induced nociception test, MECN also produced a marked and dose-dependent inhibition of PMA-induced paw licking in mice (Figure 3). The oral administration of MECN (100, 250, and 500 mg/kg) exhibited 25.33%, 36.14%, and 58.84% of inhibition, respectively, compared to the control group. In addition, ASA (100 mg/kg; used as a positive control) produced 54.09% of inhibition against PMA-induced nociception.

**Figure 4:**
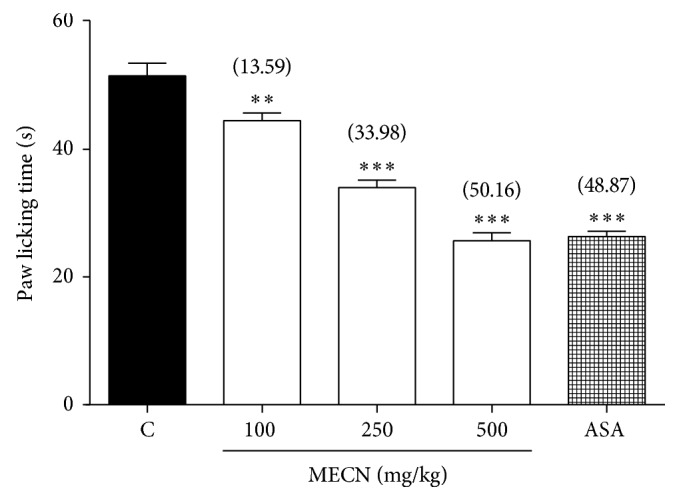
Effect of MECN on bradykinin-induced nociception in mice. Animals were treated with vehicle (10 mL/kg, p.o.), ASA (100 mg/kg, p.o.), or MECN (100, 250, and 500 mg/kg, p.o.) 60 mins before intraplantar administration of bradykinin (10 nmol/paw prepared in normal saline; 20 *µ*L) into the right hind paw. Each column represents the mean ± SEM of six mice. Statistical analyses were performed using 1-way ANOVA followed by Dunnett's post hoc test. ^∗∗∗^*p* < 0.001 compared to the control group. Values in parentheses denote percentage of inhibition.

As shown in Figure 4, MECN, when given orally, produced significant (*p* < 0.001) inhibition in a dose-dependent manner on the nociceptive caused by i.pl injection of bradykinin in mice. Pretreatment with MECN at the doses of 100, 250, or 500 mg/kg reduced the paw-licking response by 13.59%, 33.98%, and 50.16%, respectively, compared to the control group. Under similar conditions, ASA (100 mg/kg) was used as reference drug, produced 48.87% of inhibition against bradykinin-induced nociception.

### 3.2. Effects of Noradrenergic, Serotonergic, Adenosinergic, Dopaminergic, Cholinergic, and Opioidergic Systems' Inhibition on the Antinociceptive Effect of MECN

**Figure 5:**
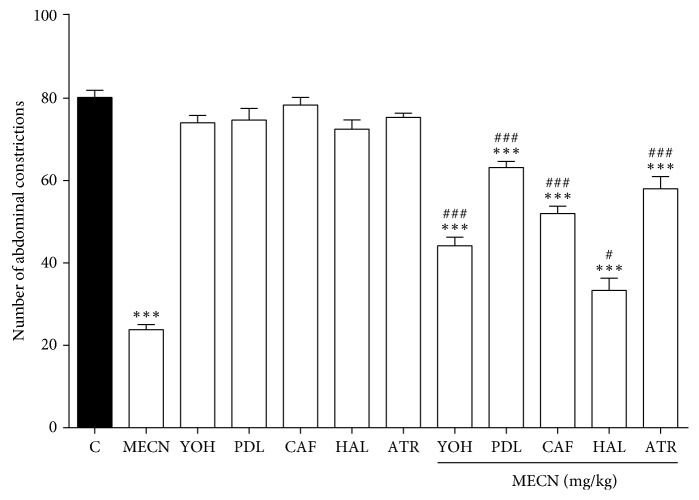
The involvement of various nonopioid receptor antagonists on MECN-induced antinociception in the acetic acid-induced abdominal constriction test in mice. Yohimbine (YOH; 0.15 mg/kg, i.p.), pindolol (PDL; 1 mg/kg, i.p.), caffeine (CAF; 3 mg/kg, i.p.), and haloperidol (HAL; 0.2 mg/kg; i.p.) were administrated 15 min before vehicle (10 mL/kg, p.o.) or MECN (500 mg/kg, p.o.). Each column represents the mean ± SEM of six mice. Statistical analyses were performed using 1-way ANOVA followed by Dunnett's post hoc test. ^∗∗∗^*p* < 0.001 compared to the control group. ^#^*p* < 0.05 and ^###^*p* < 0.001 compared to 500 mg/kg MECN-treated group.

The results depicted in Figure 5 show that pretreatment with the *α*2-adrenoreceptor antagonist YOH (0.15 mg/kg, i.p.), the 5-HT_1A/1B_ receptor antagonist PDL (1 mg/kg, i.p.), the nonselective adenosinergic receptor antagonist CAF (3 mg/kg, i.p.), the nonselective dopaminergic system antagonist HAL (0.2 mg/kg, i.p.), and the muscarinic cholinergic antagonist ATR (10 mg/kg, i.p.) significantly antagonised (*p* < 0.001) the MECN-induced antinociception (500 mg/kg, p.o.) against acetic acid-induced abdominal writhing in mice, respectively.

**Figure 6:**
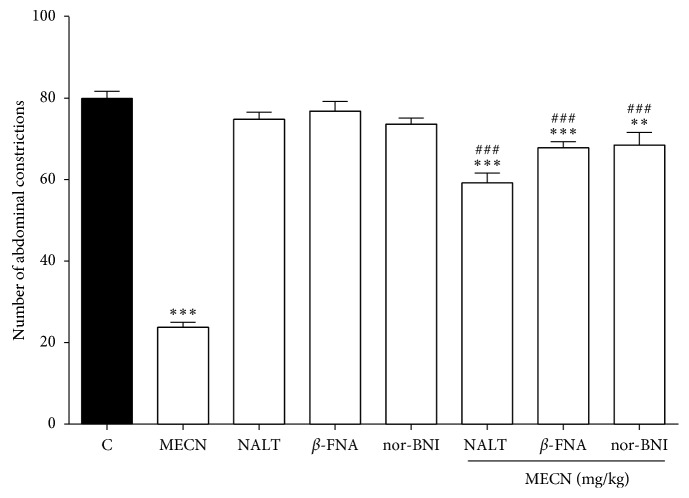
Effect of opioid receptor antagonists on MECN-induced antinociception in the acetic acid-induced abdominal constriction test in mice. *β*-funaltrexamine (*β*-FNA; 10 mg/kg, i.p.), naltrindole (NALT; 1 mg/kg, i.p.), or nor-binaltorphimine (nor-BNI; 1 mg/kg, i.p.) were administered 90 min, 15 min, and 30 min, respectively, before oral administration of vehicle (10 mL/kg) or MECN (500 mg/kg). Each column represents the mean ± SEM of six mice. Statistical analyses were performed using 1-way ANOVA followed by Dunnett's post hoc test. ^∗∗^*p* < 0.001 and ^∗∗∗^*p* < 0.001 compared to control group. ^###^*p* < 0.001 compared to 500 mg/kg MECN-treated group.

Consequently, as demonstrated in Figure 6, pretreatment with the selective *δ*-opioid receptor antagonist NALT significantly reversed (*p* < 0.001) the antinociceptive effect of MECN (500 mg/kg, p.o.). Thus, similar results obtained by pretreatment with the selective *μ-*opioid receptor antagonist *β*-FNA and *κ*-opioid receptor antagonist nor-BNI significantly reversed (*p* < 0.001) the antinociceptive effects of MECN against acetic acid-induced abdominal writhing in mice, respectively.

### 3.3. Effect of Different Potassium Channel Blockers on the Antinociceptive Effect of MECN

**Figure 7:**
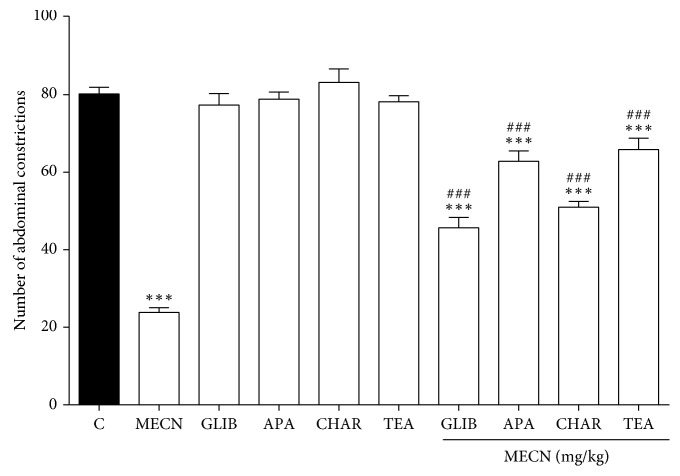
Effect of glibenclamide, apamin, charybdotoxin, and tetraethylammonium chloride on MECN-induced antinociception in the acetic acid-induced abdominal constriction test in mice. Animals were pretreated with glibenclamide (GLIB; 10 mg/kg, i.p.), apamin (APA; 0.04 mg/kg, i.p.), charybdotoxin (CHAR; 0.02, i.p.), or tetraethylammonium chloride (TEA; 4 mg/kg, i.p.) 15 min before oral administration of either vehicle (10 mL/kg) or MECN (500 mg/kg). Each column represents the mean ± SEM of six mice. Statistical analyses were performed using 1-way ANOVA followed by Dunnett's post hoc test. ^∗∗∗^*p* < 0.001 compared to control group. ^###^*p* < 0.001 compared to 500 mg/kg MECN-treated group.

As shown in Figure 7, pretreatment with ATP-sensitive K^+^ channel inhibitor GLIB (10 mg/kg, i.p.), inhibitor of small conductance Ca^2+^-activated K^+^ channels APA (0.04 mg/kg, i.p.), inhibitor of large conductance Ca^2+^-activated K^+^ channels CHAR (0.02 mg/kg, i.p.), and non-selective voltage-dependent K^+^ channel inhibitor TEA (4 mg/kg, i.p.) significantly reversed (*p* < 0.001) the antinociceptive effect of MECN (500 mg/kg, p.o.) in the acetic acidinduced abdominal writhing test.

**Figure 8:**
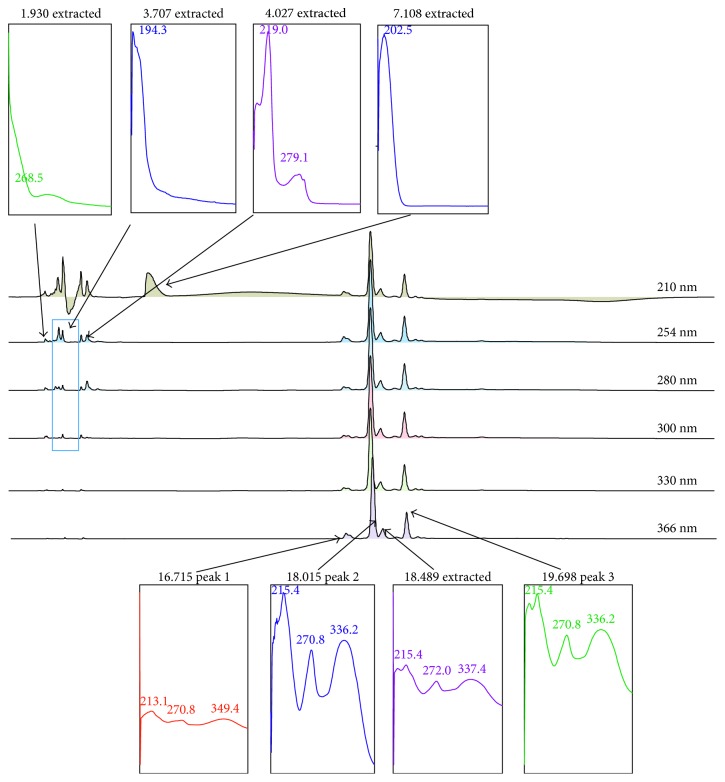
HPLC profile of MECN. Eight peaks were detected from the chromatogram of MECN at various wavelengths ranging between 210 and 366 nm. Interestingly, four peaks were detected at 366 nm, and each of them possessed the maximum wavelength (*λ*_max_) value that represents flavonoid-based bioactive compounds. Comparison between chromatogram obtained from MECN against several pure flavonoids, namely, fisetin, quercetin, rutin, quercitrin, naringenin, genistein, pinostrobin, hesperetin, and dihydroquercetin, at 366 nm shows that none of the peaks of MECN matched any of the flavonoids peak.

Figure 8 shows the chromatogram profile of MECN following the HPLC analysis carried out at the respective condition described earlier. Approximately 8 peaks were identified at various wavelengths (210–366 nm). Those peaks were detected at the retention time (*R*_*T*_; min) of 1.930 (P1), 3.707 (P2), 4.027 (P3), 7.108 (P4), 16.715 (P5), 18.015 (P6), 18.489 (P7), and 19.698 (P8) mins. Of these, peaks P5, P6, P7, and P8 were detected at the wavelength of 366 nm. Further analysis of all peaks demonstrated that the 8 peaks were detected at the maximum wavelength (*λ*_max_) ranging between 268.5 (P1), 194.3 (P2), 219.0–279.1 (P3), 202.5 (P4), 213.1–270.8–349.4 (P5), 215.4–270.8–336.2 (P6), 215.4–272.0–337.4 (P7), and 215.4–270.8–336.2 (P8) nm, respectively.

### 3.4. HPLC, UHPLC-ESI, and GCMS Profiling of MECN

Further comparison made between the chromatogram of MECN against the chromatogram of several standard compounds revealed that none of the pure compounds' peak matched the peaks of MECN, which indicates their absence in the extract (data not shown).

Table 1 shows the list of several flavonoid-based bioactive compounds with reported antinociceptive activity that has been previously identified in MECN following the UHPLC-ESI analysis.

Table 2 shows the list of several volatile bioactive compounds with reported antinociceptive activity that has been previously identified in MECN using the GCMS procedure.

## 4. Discussions

Previous study reported that the oral systemic administration of methanol extract of *C. nutans* (MECN) exerts significant antinociceptive in both chemical (acetic acid and formalin)-induced and thermal (hot plate)-induced nociception test models [12]. Overall, these findings indicate that the antinociceptive activity of MECN is mediated through the central and peripheral mechanisms. Moreover, MECN was also reported to exert the antinociceptive activity via activation of opioid receptors and modulation of the L-arginine/NO-dependent/cGMP-independent pathway [12].

In the present study, the possible mechanisms of antinociception of MECN were further investigated. The results obtained revealed that the antinociceptive activity of MECN was (i) able to inhibit the nociceptive response invoked by the respective i.pl administration of capsaicin, glutamate, PMA, and bradykinin, hence suggesting the respective modulation of vanilloid receptors, inhibition of NMDA/non-NMDA receptors or NO and NO-related substance release, phosphorylation of PKC-activated vanilloid receptor pathway, and inhibition of B_2_ receptor activation; (ii) inhibited by pretreatment with several nonopioid receptor inhibitors, namely, YOH, PDL, CAF, HAL, and ATR, thus suggesting its ability to inhibit the respective *α*2-adrenergic, serotonergic, adenosinergic, dopaminergic, or muscarinic cholinergic receptor systems; (iii) inhibited by pretreatment with inhibitors of different subtypes of opioid receptors, namely, *β*-FNA, nor-BNI, and NALT, hence implying the extract ability to inhibit the respective *μ*-, *κ*-, and *δ*-opioid receptors; and (iv) inhibited by pretreatment with various inhibitors of K^+^ channels, namely, GLIB, APA, CHAR, and TEA, thus suggesting the respective involvement of ATP-sensitive, small conductance Ca^2+^-activated, large conductance Ca^2+^-activated, and nonselective voltage-dependent K^+^ channels in the modulation of MECN antinociception.

The involvement of capsaicin receptors, also known as transient receptor potential vanilloid type 1 (TRPV1) receptors, in the modulation of nocicpetive transmission has been well documented [32]. The painful sensations provoked by capsaicin are subsequent to its binding TRPV1, a nonselective cation channel that prefers calcium. Other than capsaicin, this receptor is stimulated by noxious temperatures with a threshold *in vitro* of >43°C, protons and anandamide to name a few. Based on the threshold of activation mentioned above, it is suggested that TRPV1 is inactive at normal body temperature via dynamic regulation and is significantly lowered during inflammation. TRPV1 activity is positively regulated inside the cell after phosphorylation by protein kinases and ultimately gives rise to a complex series of events collectively referred to as neurogenic inflammation. Some activators such as prostaglandins and bradykinin modulate the activity of the receptor indirectly by activating different protein kinases inside the cell. Interestingly, Caterina et al. [33] have earlier demonstrated that mice lacking TRPV1 receptor showed normal responses to noxious mechanical stimuli but exhibited no vanilloid-evoked pain behavior. Moreover, these mice were also impaired in the detection of painful heat and showed little thermal hypersensitivity in the setting of inflammation. These findings indicate the important role of TRPV1 receptors in the modulation of different modalities of pain sensation and for tissue injury-induced thermal hyperalgesia.

The role of glutamate, a major excitatory neurotransmitter, in the nociceptive processes in both acute and chronic pain has been well-documented [34]. Glutamate, widely distributed in the entire nervous system and also colocalized with its receptors in areas of the brain, spinal cord, and periphery that are involved in pain sensation and transmission, mediates its effects via two broad types of receptors: ionotropic and metabotropic [35]. The primary afferent fibers that convey sensory, including nociceptive information from the periphery to the dorsal horn, are now known to use glutamate as the primary transmitter. Concurrent with the above statement, application of glutamate to the spinal cord or periphery induces nociceptive behaviors while inhibition of glutamate release, or of glutamate receptors, in the spinal cord or periphery attenuates both acute and chronic pain in animal models. Thus, glutamate and glutamate receptors play a crucial role in perception and integration of nociceptive signals and in their relay to supraspinal centers [35]. The involvement of glutamatergic system in the nociceptive neurotransmission, at the peripheral, spinal, and supraspinal levels, has been very much acknowledged to involve modulation via the N-methyl-D-aspartate (NMDA) and non-NMDA receptors, as well as by the release of NO and NO-related substances [34]. Meanwhile, NMDA receptor antagonists have been proven to inhibit the spread of pain sensation and to reduce the hyperexcitability of spinal cord neurons triggered by C-fiber stimulation. Other than that, several studies have also demonstrated that attenuation of glutamate-induced nociceptive response could be attributed to the inhibition of PKC pathway [36]. PKC has been shown to indirectly sensitize the central glutamate-associated NMDA receptors located in the postsynaptic neuron [37]. Taking into consideration the ability of MECN to attenuate glutamate-induced nociception, it is plausible to propose the involvement of glutamatergic system in the antinociceptive activity of MECN possibly via modulation of NMDA- and PKC-related pathways to name a few.

Protein kinase C (PKC) is an important regulator of various cellular functions, and various studies have successfully demonstrated the role of PKC in the nociceptive transmission at the central and peripheral levels [38]. The enzyme has been known to localize in both peripheral and central nervous system sites that process pain and is able to phosphorylate several cellular components that serve as key regulatory components in signal transduction pathways of nociceptor excitation and sensitization [39]. The activation of PKC occurs through interaction with intracellular lipid second messengers phosphatidylserine and diacylglycerol (DAG), and high level of calcium ions [40], which leads to the phosphorylation of many cellular components including TRPV1, NMDA, and bradykinin receptors [41–43]. Rosenbaum and Simon [44] have suggested that phosphorylation of TRPV1 receptor by PKC caused not only potentiation of capsaicin- or proton-evoked response, but also reduces the temperature threshold for TRPV1 receptor activation. On contrary, the inhibitors of PKC have been reported to not only prevent the phosphorylation of TRPV1 receptor, but also reducing the sensitization of this capsaicin-sensitive receptor, which makes it less responsive to agonist action [45]. Concurrent with these reports, the present study has also demonstrated the ability of MECN to reverse the nociceptive effect of PMA, an activator of PKC, indicating the extract potential to inhibit the PKC-modulated pathways. The extract ability to inhibit PKC-modulated nociception might also be linked to its ability to inhibit the capsicin-induced TRPV1-modulated nociception as described above. Other than that, PKC has been reported to indirectly involve in the central sensitization of normally silent N-methyl D-aspartate (NMDA) glutamate receptors located in the postsynaptic neuron suggesting that the activation of PKC also plays an important role in the nociceptive transmission through glutamatergic system [16, 17]. Thus, the ability of MECN to attenuate capsaicin-induced/PKC-modulated nociception was in agreement with the ability of MECN to inhibit glutamate-induced nocicpetion as described earlier. These findings seem to suggest the ability of MECN to modulate the NMDA receptor-mediated nociceptive transmission.

Bradykinin, a potent inflammatory peptide messenger, is released from damage tissues during neurogenic inflammation and has been reported to cause peripheral sensitization in the PNS and central sensitization in the CNS [46]. At the peripheral level, bradykinin sensitizes nociceptor peripheral terminals by acting on the A*δ*- and C-fibers and evokes the release and synthesis of other second messengers, including prostaglandins, nitric oxide, and neurokinins, thus reducing pain threshold. Bradykinin preferentially acts at the B_2_ receptors, which are largely constitutive, being present at a relatively constant density on various cells including nociceptive primary afferent neurons, thus causing a direct activation of PKC-signaling pathway and an indirect activation of the PKA-signaling pathway [47]. At the central level, bradykinin is released in the spinal cord in response to nociceptor inputs and acts as a synaptic neuromodulator, potentiating glutamatergic synaptic transmission to produce pain hypersensitivity via the activation of PKC pathway. Recent study has reported that bradykinin-evoked pain hypersensitivity is NMDA receptor-dependent and that central sensitization is B_2_ receptor-dependent [48]. Based on these reports and the present observations that MECN inhibited bradykinin-induced nociception, it is plausible to suggest that MECN induced antinociceptive activity possibly via its nonselective inhibition of B_2_ receptors, TRPV1 receptors, or PKC-mediated pathway.

Receptors are nerve endings that are located in the defined area or respective field from which they receive information. They respond to noxious stimuli and transmit the information via afferent or sensory fibers to the CNS. Through pharmacological exploitation, pain can be altered through the manipulation of pain transmission and inhibitory signals within the nociceptive pathway. The transmission of pain to the brain can be decreased or the inhibitory signals from the brain can be increased in order to achieve pain relief. In an attempt to identify the basic mechanism of antinociception of compound/extract, a range of studies have exploited the pharmacological tools such as various types of receptor antagonists [18, 49–51]. In order to understand the mechanisms via which the compound/extract is working, the compound/extract was first pretreated with the respective receptor antagonist (receptor antagonist + compound/extract), and the percentage of antinociceptive effect observed using any of the nociceptive models was compared with the percentage of antinociceptive effect seen after the compound/extract was pretreated with distilled water (dH_2_O; dH_2_O + compound/extract). The receptors were usually divided into two classes, namely, opioid and nonopioid receptors, when discussing about pain. In the previous study, MECN has been reported to exert antinociceptive activity via the activation of opioid receptors based on the observation that MECN-produced antinociceptive activity was inhibited by naloxone, a non-specific opioid antagonist [12]. In the present study, the role of specific subtypes of opioid receptors, namely, *μ*-, *κ*-, and *δ*-opioid receptors, in the modulation of MECN antinociception has been proven. Interestingly, inhibition of the three subtypes of opioid receptors reduces the antinociceptive efficacy of MECN suggesting that the extract exerts a nonselective opioid action as seen with bremazocine and buprenorphine [52, 53].

Nonopioid analgesics are among the most widely used treatments for pain [54]. Other than the inhibition of COX and prostaglandin synthesis, accumulating evidence has demonstrated the multiple actions of analgesics with other systems during pain. These systems, classified as nonopioid receptor systems, include interaction with the monoaminergic pathways, such as noradrenergic, cholinergic, and serotonergic systems, or the purinergic pathway, such as adenosinergic system. The quest to find new/novel nonopioid-mediated analgesics is emerging and can help to overcome the side effects associated with the use of opioid analgesics especially psychological addiction, abuse, diversion of uses, dependence, and tolerance [55]. The involvement of noradrenergic system in nociception at spinal and supraspinal levels has been proven to be mediated through activation of *α*-adrenoceptors and descending inhibitory pathways [56]. Yohimbine, the *α*_2_ adrenoceptor antagonist, has been reported to antagonize the antinociceptive effects of nonopioid octacosanol when assessed using the mouse abdominal constriction test [57]. Similarly, yohimbine also inhibited the antinociceptive activity of MECN when assessed using the same assay, hence suggesting that the antinociceptive activity of MECN is partly mediated via the *α*_2_ adrenergic receptor activation. Meanwhile, previous studies have also reported on the participation of activated *β*-adrenergic receptors in central and peripheral nociceptive transmission [58, 59]. In the present study, MECN-exerted antinociceptive activity was inhibited by pindolol, thus suggesting the modulation of MECN antinociceptive activity occurs partly via the activation of *β-*adrenergic receptors.

Adenosine, derived from degradation of adenosine triphosphate (ATP) via ectonucleotidase pathway [60], activates the adenosine receptors, which appears to partly involve in the modulation of nociceptive and inflammatory pathways [61]. Caffeine has earlier been reported to inhibit various adenosine receptors, namely, A_1_, A_2A_, A_2B_, and A_3_ receptors [62]. Activation of A_1_ receptors, in particular, has been shown to cause antinociceptive activity via the reduction in PGE_2_ synthesis/activity [63]. Moreover, several reports have demonstrated that drugs with ability to increase monoamine availability or to act through the activation of opioid receptors exert antinociceptive activity via the activation of adenosine receptors [64, 65]. Correspondingly, MECN antinociceptive activity was inhibited by caffeine, a nonselective adenosinergic receptor antagonist, indicating that the extract attenuated the nociceptive transmission partly through the activation of adenosinergic receptors. Interestingly, the ability of MECN to attenuate nociceptive transmission via the modulation of adenosinergic and serotonergic systems as demonstrated in the present study is concurrent with report made by [66]. On the other hand, blockade of the A1 receptors has been shown to involve modulation of the NO/cGMP/KATP pathway [63] which is inconsistent with our previous report on the ability of MECN to modulate NO-dependent and cGMP-independent pathways [12]. Similarly, NO regulation of inflammation has frequently been associated with signal transduction events that do not involve cGMP [67, 68]. However, the regulatory functions of nitric oxide (NO) that bypass the second messenger cGMP have been reported but are incompletely understood [69]. Interestingly, a report by Morioka et al. [70] can be used to suggest one of the possible mechanisms involving the cGMP-independent pathway via which MECN might partly exert antinociceptive activity. The report revealed that NO enhances the IL-1*β*-induced increase in COX-2 expression in cultured dorsal root ganglion cells via a cGMP-independent-mediated pathway leading to facilitation of Substance P release. This interaction observed between NO and COX in primary afferent neurons might contribute to the change in nociceptive perception in inflammatory hyperalgesia.

The role of dopaminergic neurotransmission in the modulation of pain perception has been previously discussed [71]. Dopamine has been reported to be pronociceptive at peripheral D_1_- and D_2_-like receptors or at other locations in the CNS while recent study has suggested that the peripheral D_1_-like receptors to play role in peripheral sensitization [72]. On the other hand, dopamine exerts antinociceptive effects at D_2_-like receptors located centrally in the trigeminocervical complex, which is activated following the peripheral primary afferent excitation [73]. However, this separation remains as a matter for discussion. In the present study, pretreatment with haloperidol, a dopamine D_2_ receptor antagonist was also found to inhibit MECN-produced antinociceptive activity, which indicates the involvement of dopaminergic receptor system in the extract's antinociceptive activity and the possible role of MECN as D_2_ receptor agonists. Other than that, a close relationship between the opioid and dopaminergic systems has been proven by various researches [74–76]. These reports were concurrent with the present study, which demonstrated the involvement of opioidergic and dopaminergic systems in the modulation of MECN-exerted antinociceptive activity. Based on the previous studies, it is plausible to propose that the mechanisms of antinociception adopted by MECN might involve (i) induction of endogenous opioids release [77] or (ii) direct activation of the dopaminergic system [78].

Acetylcholine, a neurotransmitter found in both the PNS and CNS of humans, plays a role in the inhibition and regulation of the pain transmission [1]. The physiological effects of acetylcholine, released from peripheral sources following cutaneous injury, can activate sensory afferents through muscarinic receptors as well as nicotinic receptors. According to Bektas et al. [1], the transmission of pain impulses may be suppressed via activation of mAChRs that are located on peripheral nociceptors of the skin. Muscarinic receptors activation contributes to the release of various modulators and to the change of various ion channels permeability, which in turn provides of both direct and indirect control in pain modulation [79]. Some research reports that cholinergic agonists also have analgesic effects on animal experiments via cholinergic stimulation and following spinal mAChRs activation [80]. In the present study, atropine, a nonselective muscarinic antagonist, was found to attenuate the antinociceptive activity of MECN, thus suggesting the involvement of muscarinic receptors in the extract's antinociception. Interestingly, muscarinic receptors have also been shown to mediate the analgesic effect of other analgesic agents, including morphine [81]. This finding supported the present observation that MECN possesses opioid- and muscarinic-mediated antinociceptive activity.

Ion channels are essential for controlling neuronal excitability, which is one of the steps in generation of most pain signals in human nervous system. Electrical excitation starts in the peripheral somatosensory nerves and is controlled by an intricate set of ion channels that are coordinated to produce a degree of excitation that is proportional to the strength of the external stimulation [82]. K^+^ channels are the most populous, widely distributed, and diverse class of ion channels in neurons. Upon activation, K^+^ channels facilitate an extremely rapid transmembrane K^+^ efflux that can influence action potential threshold, waveform, and frequency. Because K^+^ channel opening repolarizes (or even hyperpolarizes) the neuronal membrane, this function can limit action potential generation and firing rate. A variety of antinociceptive drugs mediate their action by directly opening spinal K^+^ channels [83]. In the present study, several K^+^ channels' blockers, namely, GLIB (an inhibitor of ATP-sensitive K^+^ channels), APA (an inhibitor of small conductance Ca^2+^-activated K^+^ channels), CHAR (an inhibitor of large conductance Ca^2+^ -activated K^+^ channels), and TEA (an inhibitor of nonselective voltage dependent K^+^ channel), have been shown to inhibit the antinociceptive activity of MECN. The ability of GLIB, APA, CHAR, and TEA to inhibit the antinociceptive activity of MECN is concurrent with report made by Longhi-Balbinot et al. [84] on the ability of the same K^+^ channel blockers to inhibit the antinociceptive effect of morphine. The ability of MECN to trigger different types of K^+^ channels could also be attributed to the fact that MECN is a crude extract, which has been shown to contain a mixture of several volatile and nonvolatile phytoconstituents [12]. These phytoconstituents might act alone or synergistically to activate different types of K^+^ channels leading to the observed antinociceptive activity.

Several classes of phytoconstituents have been identified in MECN such as flavonoids, saponins, triterpenes, and steroids [12]. In general, flavonoids [85, 86], saponins [87, 88], and triterpenes [89, 90] have been widely reported to exert antinociceptive activity. Thus, it is plausible to suggest that these different classes of bioactive compounds might act synergistically or collectively to produce the observed antinociceptive activity of MECN. In addition, MECN has also been subjected to phytochemical analysis using the UHPLC-ESI and GC-MS methods. At least 16 phenolic compounds were identified using the UHPLC-ESI method, and, of these, several phenolic compounds, namely, gallic acid, caffeic acid, ferulic acid, vitexin, orientin, luteolin, apigenin, have been reported to exert antinociceptive activity [21–28]. On the other hand, GC-MS analysis of MECN demonstrated the presence of at least 39 volatile bioactive compounds of which only *α*-linolenic acid, phytol, and *n*-hexadecanoic acid were reported to demonstrate antinociceptive activity [29–31]. The presence of different types of bioactive compounds with antinociceptive activity in MECN might explain the ability of MECN to demonstrate antinociceptive activity via activation of various mechanisms.

## 5. Conclusions

In conclusion, the present study has demonstrated the possible mechanisms of antinociception of MECN to involve (i) activation of the opioidergic receptors; (ii) activation of the nonopioidergic systems, namely, noradrenergic, serotonergic, adenosinergic, dopaminergic, and muscarinic-cholinergic receptors; (iii) opening of different types of K^+^ channels such as ATP-sensitive and K^+^-channels, voltage-activated K^+^ channels, and Ca^2+^-activated K^+^ channels; and (iv) inhibition of PKC-, bradykinin-, glutamate-, and TRPV1-mediated nociceptive pathways.

## Figures and Tables

**Table 1:** Several flavonoid-based bioactive compounds with antinociceptive potential that has been previously identified from MECN using the UHPLC-ESI procedure.

Number	Flavonoid-based bioactive compounds	Antinociceptive model used	Antinociceptive activity reported by
(1)	Gallic acid	(i) Inflammatory pain model	Trevisan et al. [21]
		(ii) Carrageenan-induced hyperalgesia test	
		(iii) Neuropathic pain model	
		(iv) Chronic constriction injury (CCI)-induced neuropathic pain test	
		(v) Cold allodynia test	
		(vi) Mechanical allodynia test	

(2)	Caffeic acid	(i) Acetic acid-induced writhing test	Mehrotra et al. [22]
		(ii) Formalin-induced paw licking test	
		(iii) Hot plate test	
		(iv) Tail flick test	

(3)	Ferulic acid	(i) Neuropathic pain model	Xu et al. [23]
		(ii) CCI-induced neuropathic pain test	
		(iii) Mechanical allodynia test	
		(iv) Thermal hyperalgesia	
		(v) Electronic von Frey test	
		(vi) Hot plate test	

(4)	Vitexin	(i) Mechanical hyperalgesia test	Zhu et al. [24]
		(ii) Electronic von Frey test	
		(iii) Acetic acid-induced writhing test	
		(iv) Capsaicin-induced nociceptive test	Borghi et al. [25]
		(v) Formalin test	

(5)	Orientin	(i) Acetic acid-induced writhing test	Da Silva et al. [26]
		(ii) Capsaicin-induced nociceptive test	
		(iii) Glutamate-induced nociceptive test	

(6)	Luteolin	(i) CCI-induced neuropathic pain test	Hara et al. [27]
		(ii) Mechanical hyperalgesia	
		(iii) Electronic von Frey test	
		(iv) Thermal hyperalgesia	
		(v) Hot plate test	
		(vi) Cold hyperalgesia	
		(vii) Cold plate test	

(7)	Apigenin	(i) Acetic acid-induced writhing test	Pinheiro et al. [28]
		(ii) Formalin-induced paw licking test	
		(iii) Hot plate test	

**Table 2:** Several volatile bioactive compounds with reported antinociceptive activity that has been previously identified in MECN using the GCMS procedure.

Number	Flavonoid-based bioactive compounds	Antinociceptive model used	Antinociceptive activity reported by
(1)	9,12,15-octadecatrienoic acid (*α*-linolenic acid)	(i) Acetic acid-induced writhing test	Ren and Chung [29]
		(ii) Randal–Selitto assay	

(2)	Phytol	(i) Acetic acid-induced writhing test	Santos et al. [30]
		(ii) Formalin-induced paw licking test	
		(iii) Hot plate test	

(3)	*n*-hexadecanoic acid	(i) Neuropathic pain model	Aparna et al. [31]
		(ii) CCI-induced neuropathic pain test	
		(iii) Mechanical allodynia test	
		(iv) Thermal hyperalgesia	
		(v) Electronic von Frey test	
		(vi) Hot plate test	
